# Trained immunity in diabetes: emerging targets for cardiovascular complications

**DOI:** 10.3389/fendo.2025.1533620

**Published:** 2025-05-14

**Authors:** Yanan Bai, Jianglan Wu, Weixiong Jian

**Affiliations:** ^1^ College of Traditional Chinese Medicine, Hunan University of Traditional Chinese Medicine, Changsha, Hunan, China; ^2^ Diagnostics of Traditional Chinese Medicine, National Key Discipline, Hunan University of Traditional Chinese Medicine, Changsha, Hunan, China

**Keywords:** diabetes, atherosclerosis, trained immunity, metabolism, inflammation, epigenetics

## Abstract

Diabetes is a metabolic disorder primarily characterized by persistent hyperglycemia. Diabetes-induced inflammation significantly compromises cardiovascular health, greatly increasing the risk of atherosclerosis. The increasing prevalence of harmful lifestyle habits and overconsumption has contributed substantially to the global rise in diabetes-related cardiovascular diseases, creating a significant economic and healthcare burden. Although current therapeutic strategies focus on blood glucose control and metabolic regulation, clinical observations show that diabetic patients still face persistent residual risk of AS even after achieving metabolic stability. Recent studies suggest that this phenomenon is linked to diabetes-induced trained immunity. Diabetes can induce trained immunity in bone marrow progenitor cells and myeloid cells, thus promoting the long-term development of AS. This article first introduces the concept and molecular mechanisms of trained immunity, with particular emphasis on metabolic and epigenetic reprogramming, which plays a crucial role in sustaining chronic inflammation during trained immunity. Next, it summarizes the involvement of trained immunity in diabetes and its contribution to AS, outlining the cell types that can be trained in AS. Finally, it discusses the connection between diabetes-induced trained immunity and AS, as well as the potential of targeting trained immunity as an intervention strategy. Understanding the molecular mechanisms of trained immunity and their impact on disease progression may provide innovative strategies to address the persistent clinical challenges in managing diabetes and its complications.

## 1 Introduction

Diabetes represents a persistent metabolic condition marked by increased blood glucose concentrations, which arise from inadequate insulin production, insulin resistance, or a combination of these mechanisms. The swift rise in its global occurrence has rendered it a notable issue for public health (1). A recent forecast from the International Diabetes Federation indicates that around 536.6 million adults between the ages of 20 and 79 were diagnosed with diabetes in 2021, accounting for roughly 10.5% of the worldwide population. By the year 2045, projections indicate that the population of individuals diagnosed with diabetes will increase to 783.2 million, reflecting a prevalence rate of around 12.2% (2). In China, the prevalence of diabetes exhibited a significant rise, escalating from 9.7% in 2007 to 11.2% in 2017. This trend positions the nation as having the highest number of diabetes cases and the most substantial healthcare burden on a global scale (3). Diabetes is categorized into two main forms: type 1 diabetes (T1D) and T2D. Both forms are linked to serious complications and pose a considerable challenge to public health (4).

Atherosclerosis(AS) is one of the most common complications of diabetes, and the atherosclerotic cardiovascular diseases (ASCVDs) it triggers are the leading cause of death among individuals with diabetes (5). Epidemiological studies suggest that individuals diagnosed with T1D or T2D exhibit a two to fourfold increased likelihood of developing ASCVDs (6). Prospective studies have shown that hyperglycemia, a defining characteristic of diabetes, serves as an independent and significant risk factor for cardiovascular diseases (CVDs) in individuals with diabetes (7, 8). The low-grade systemic inflammation triggered by hyperglycemia notably accelerates AS and results in significant vascular lesions (9). Furthermore, this heightened risk is intricately associated with various significant pathological mechanisms observed in individuals with diabetes, such as dyslipidemia, genetic predisposition, insulin resistance, and obesity (5, 10). The presence of these metabolic comorbidities not only heightens the likelihood of cardiovascular incidents but also intensifies the progression of diabetes, creating a detrimental feedback loop. Individuals with diabetes exhibit elevated residual risks in the management of CVDs, rendering conventional treatment approaches frequently inadequate for the comprehensive control of cardiovascular events. Research indicates that previous conditions such as dyslipidemia, hyperglycemia, or being overweight can result in enduring “legacy effects,” where the likelihood of vascular complications continues even after achieving normalized blood glucose and metabolic (11, 12). Recent findings indicate that this phenomenon could be associated with the trained immunity observed in individuals diagnosed with diabetes.

Trained immunity describes the phenomenon where innate immune cells develop memory-like responses to previous stimuli, allowing them to respond more robustly to future identical or varied challenges (13). Trained immunity presents a complex duality. On one hand, it functions as a defensive strategy against recurring infectious agents and neoplasms; conversely, when innate immune cells play a role in the pathophysiology of chronic inflammatory conditions, such as AS, trained immunity fosters enduring disease-associated characteristics and hastens disease advancement (14, 15). Recent studies have indicated the possible causal involvement of high glucose-induced activation of the innate immune response in AS, substantiating that trained immunity may serve as a mechanism connecting diabetes and AS (16). Thus, a thorough exploration of the mechanisms underlying trained immunity could yield essential new perspectives on the prevention and management of cardiovascular complications associated with diabetes.

## 2 Trained immunity

### 2.1 Brief introduction to trained immunity

**Figure 1:**
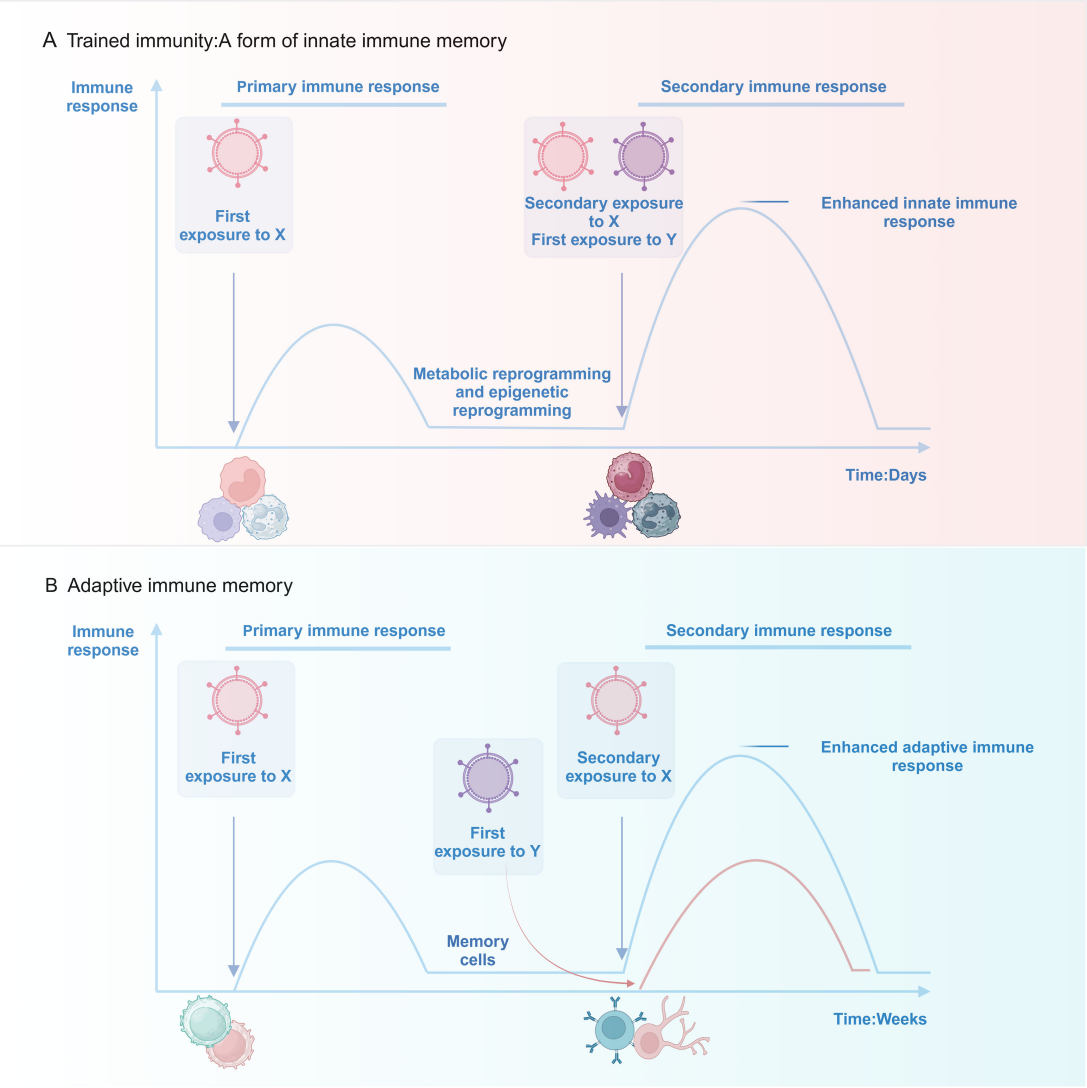
Upon exposure to stimulus X, innate immune cells (e.g., monocytes/macrophages and neutrophils) activate rapidly and develop memory via metabolic and epigenetic reprogramming. This improves their response to subsequent exposures of stimulus X or an unrelated stimulus Y. Innate immune memory can last up to a year. In contrast, adaptive immune cells (T and B lymphocytes) require weeks to form memory after activation by stimulus X, resulting in memory lasting decades. Adaptive immune memory is highly specific: re-exposure to stimulus X triggers a strong recall response, while a new stimulus Y initiates a fresh immune response.

The human immune system can be categorized into two primary components: innate immunity and adaptive immunity. The innate immune system identifies pathogen-associated molecular patterns (PAMPs) or damage-associated molecular patterns (DAMPs) via pattern recognition receptors (PRRs). This recognition triggers a swift activation of immune cells and the release of various substances, resulting in a rapid yet non-specific defense response (17). In contrast, the adaptive immune response operates at a slower pace yet produces highly specific reactions to pathogens, establishing long-term memory that enables the organism to eradicate pathogens more efficiently and effectively during subsequent exposures (18). Historically, immune memory has been regarded as a characteristic solely of adaptive immunity, whereas innate immunity was perceived as offering only non-specific defense mechanisms. In 2011, Netea et al. (13) presented the concept of “trained immunity,” demonstrating that innate immune cells possess the ability to establish enduring, non-specific memory following their initial encounter with pathogens. This leads to a more robust reaction when faced with either identical or varied stimuli, thereby questioning the conventional perspective that innate immunity is devoid of memory. The mechanisms, however, exhibit distinct characteristics compared to those of adaptive immune memory (**Figure 1**).

Trained immunity was originally believed to be triggered by external factors, including microbial infections or BCG vaccination, resulting in the enduring functional reprogramming of immune cells (19). Following the cessation of stimuli, immune cells persist in a “trained” condition, facilitating a more rapid and robust response upon subsequent exposure (20). Recent investigations have demonstrated that endogenous molecules, including oxidized low-density lipoprotein (oxLDL) (21), aldosterone (22), high glucose (16),and catecholamines (23), can also induce trained immunity. This internal stimulation could transition the innate immune system from a protective to a more hostile function, which may result in overactivation of the immune response, fostering persistent sterile inflammation, and playing a role in the emergence of chronic inflammatory conditions (24).

Trained immunity was initially recognized in myeloid cells, commonly referred to as peripheral trained immunity (25). Nonetheless, its duration (extending over months or even years) stands in stark contrast to the brief lifespan of mature myeloid cells (merely a few days). Further investigations have validated that trained immunity influences hematopoietic stem and progenitor cells (HSPCs) within the bone marrow via mechanisms of epigenetic reprogramming. (termed central trained immunity). This enables newly differentiated myeloid cells (such as monocytes, macrophages, and neutrophils) to maintain their “trained” state, thereby preserving long-term immune memory (26). Furthermore, other innate immune cells, including natural killer(NK) cells (27), innate lymphoid cells (28) and dendritic cells(DCs) (29), display comparable traits. It is important to note that trained immunity extends beyond myeloid cells; certain non-immune cells, including vascular endothelial cells (VECs) and epithelial cells (ECs), have demonstrated characteristics akin to trained immunity. This observation implies that the occurrence of this phenomenon may be more prevalent than earlier assumptions suggested (30, 31).

### 2.2 Molecular mechanisms mediating trained immunity

**Figure 2:**
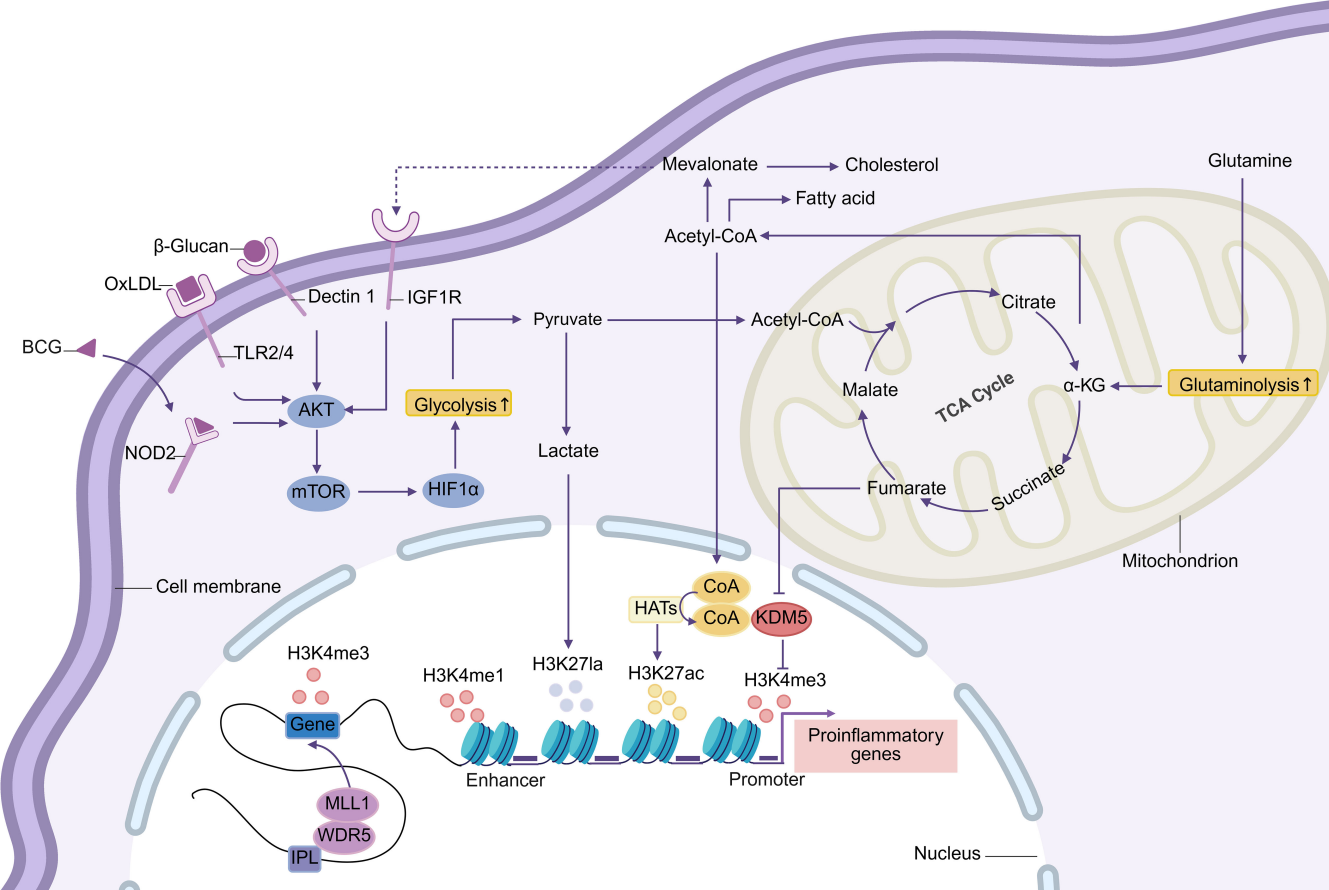
The Akt-mTOR-HIF1α pathway initiates trained immunity by triggering glycolytic reprogramming. This enhances glucose uptake and breakdown into pyruvate, which can convert to lactate or oxidize to acetyl-CoA for tricarboxylic acid (TCA) cycle citrate production. Lactate modifies histones via lactylation, while cytoplasmic citrate regenerates acetyl-CoA. Acetyl-CoA supports fatty acid/cholesterol synthesis and drives epigenetic remodeling as an HAT acetyl donor. Enhanced glutamine catabolism accumulates fumarate, inhibiting KDM5 activity. Immune gene-priming long noncoding RNAs (IPLs) gene promoters, mediating histone methylation and enhancing transcription. These metabolic-epigenetic networks form the basis of trained immunity. Abbreviations: HAT: histone acetyltransferases; oxLDL: oxidized low-density lipoprotein; IGF1R: insulin-like growth factor 1 receptor; NOD2: nucleotide-binding oligomerization domain protein 2; TLR: Toll-like receptor; α-KG: α-ketoglutarate.

Research has confirmed that trained immunity is primarily mediated through two interconnected mechanisms: metabolic reprogramming and epigenetic modification (**Figure 2**). Different stimuli may activate specific pathways and influence the expression of distinct genes (32). Additionally, post-transcriptional regulation, such as N6-methyladenosine (m6A) modification, may also play a role in trained immunity, although its exact role remains to be verified (32). Notably, trained immunity exhibits transgenerational effects: newborns whose parents received BCG vaccination show stronger non-specific protective effects and higher survival rates following BCG vaccination, suggesting that BCG confers a synergistic transgenerational immune-enhancing effect (33).

#### 2.2.1 Metabolic reprogramming

Metabolic reprogramming serves as the fundamental process that facilitates the development of trained immunity. Upon stimulation, innate immune cells experience notable metabolic reconfiguration, which entails modifications across various pathways, such as oxidative phosphorylation (OXPHOS), fatty acids metabolism, and glutaminolysis (34). The metabolic alterations observed serve to fulfill the energy requirements necessary for the establishment of immune memory, while simultaneously modulating epigenetic modifications via intermediate metabolites. This process ultimately improves the cells’ ability to respond to future stimuli (25).

Glycolysis serves as the pivotal metabolic center in the context of trained immunity. Glucose is metabolized into pyruvate, which can subsequently be transformed into lactic acid or proceed into the tricarboxylic acid (TCA) cycle (35). The hallmark “aerobic glycolysis” (Warburg effect) in immune cells enables them to preferentially produce lactic acid under normoxic conditions, a process that is not confined to hypoxic environments (36). In their resting state, immune cells rely on OXPHOS for energy, a more efficient but slower process (37). Upon activation, they rapidly switch to aerobic glycolysis to meet increased energy needs, a metabolic shift crucial for the establishment of trained immunity (38). From a mechanistic perspective, β-glucan enhances aerobic glycolysis in monocytes through the Akt-mTOR-HIF1α signaling pathway, and the inhibition of this pathway abolishes the trained immunity phenotype (39). In a comparable manner, the activation of glycolysis is crucial in the trained immunity elicited by BCG and oxLDL, underscoring the fundamental role of glycolysis in innate immune memory (21, 40). Beyond its role in energy provision, glycolysis may also contribute to immune regulation through the following mechanisms: 1) The upregulation of glycolysis increases the NAD+/NADH ratio, thereby reducing Sirtuin-1 histone deacetylase activity. *In vitro* addition of the Sirtuin-1 activator resveratrol prevents β-glucan-induced trained immunity in monocytes, highlighting the negative regulatory function of Sirtuin-1 in trained immunity (39). 2) Glycolysis supplies substrates for the pentose phosphate pathway (PPP), which generates nucleotides and NADPH. However, inhibiting the oxidative branch of PPP does not impair the induction of trained immunity, suggesting its dispensability in this context (41, 42).

The conventional view of lactic acid as merely the end product of glycolysis has been fundamentally revised. Growing evidence underscores its multifaceted roles in immune regulation. LPS stimulation induces lactic acid accumulation in bone marrow-derived macrophages (BMDMs), triggering lactylation through binding to histone lysine residues (43). This novel post-translational modification does not directly suppress early inflammatory responses but functions as a “lactate clock”, initiating after histone acetylation. It subsequently activates the homeostatic genes of M2 anti-inflammatory macrophages during the later stages of acute inflammation (44). Through epigenetic regulation, lactylation orchestrates the gene expression network during the resolution of inflammation. Although this mechanism primarily contributes to the resolution of inflammation, its role in “trained immunity” macrophages, which require sustained activation, is limited. Recent breakthroughs, however, have unveiled a dual-pathway regulatory mechanism of lactic acid in trained immunity: metabolically, lactic acid is catalyzed by lactate dehydrogenase A (LDHA) to generate acetyl-CoA, which exhibits greater mitochondrial oxidation efficiency than glucose, supporting the energy needs of innate immune cells via enhanced ATP synthesis; epigenetically, lactylation enhances chromatin accessibility, directly activating the transcription of inflammatory cytokines such as IL-6 and TNF-α (44). This process depends on LDHA and monocarboxylate transporter 1 (MCT1). Blocking either component disrupts cytokine secretion and impaired anti-infection capabilities, underscoring lactic acid’s bridging role between metabolic reprogramming and epigenetic regulation. Notably, current research faces three critical limitations: first, LDHA is involved in both lactic acid metabolism and glycolysis (the latter being a hallmark of trained immunity), complicating the ability to separate the independent contribution of lactylation (44); second, the regulatory differences between lactylation in acute inflammatory resolution and the chronic inflammatory environment of trained immunity remain unclear; third, the complex interactions between lactylation and other histone modifications, such as acetylation, are yet to be fully understood. Future studies should aim to develop conditional lactylation-deficient models and incorporate multi-dimensional analyses of metabolomics and epigenomics to systematically investigate the spatiotemporal regulatory foundation of the lactic acid metabolic network. This approach will provide a solid theoretical foundation for the development of targeted immune intervention strategies focused on the lactic acid pathway.

Glutamine catabolism is another critical feature of trained immunity. In monocytes activated by BCG or β-glucan, glutamine metabolism is significantly enhanced, leading to the production of glutamate and α-ketoglutarate (α-KG). The latter enters the TCA cycle, contributing to the accumulation of fumarate, succinate, and malate (42). Notably, fumarate specifically inhibits histone lysine demethylase 5 (KDM5), increasing the level of H3K4me3 at the promoters of pro-inflammatory genes and facilitating epigenetic reprogramming (42). Moreover, fumarate stabilizes HIF1α protein, thereby maintaining glycolytic flux (45). Additionally, α-KG serves as a cofactor for TET demethylases and JMJD3 histone demethylases (KDM6), contributing indirectly to epigenetic regulation (46, 47). Collectively, these findings outline a critical mechanism by which metabolic intermediates regulate epigenetic modifications to establish trained immunity.

Acetyl-CoA functions as a central node in the metabolic regulation of trained immunity. Within mitochondria, pyruvate is converted into acetyl-CoA, which enters the TCA cycle. Subsequently, citrate is transported to the cytoplasm, where it is re-synthesized into acetyl-CoA via the ATP-citrate lyase (ACLY) (48, 49). This crucial metabolite regulates trained immunity through three mechanisms: 1) it participates in fatty acid synthesis (FAS), which is essential for aldosterone-induced trained immunity (22); 2) it is converted into mevalonate, entering the cholesterol synthesis pathway. This process critical for oxLDL and β-glucan-induced trained immunity, as statins can block it by inhibiting HMG-CoA reductase and thereby suppress trained immunity (50). Studies have shown that mevalonate promotes glycolysis by activating the IGF-1R-mTOR pathway, thereby forming a positive feedback loop during the induction of trained immunity. Its accumulation leads to an endogenous trained immunity phenotype (50); 3) as an acetyl donor for histone acetyltransferases (HATs), it contributes to the epigenetic regulation of trained immunity (51).

OXPHOS plays a stimulus-dependent role in trained immunity. BCG, oxLDL, catecholamines, and low-dose β-glucan (1 μg/ml) all enhance OXPHOS activity in monocytes (21, 23, 40, 52). Low-dose β-glucan achieves this by upregulating H3K4me1 levels at enhancer regions of TCA cycle enzyme genes via Set7 methyltransferase (52). Conversely, high-dose β-glucan (10 μg/ml) inhibits OXPHOS (39), and the underlying dose-dependent mechanism requires further investigation. Notably, the OXPHOS inhibitor oligomycin blocks oxLDL and β-glucan-induced trained immunity, but does not affect the BCG model (21, 41, 52), suggesting that different stimuli may activate divergent metabolic pathways.

#### 2.2.2 Epigenetic reprogramming

Epigenetic reprogramming dynamically alters chromatin structure and accessibility, facilitating gene expression remodeling, which is a central mechanism in the establishment of trained immunity. During initial stimulation, innate immune cells undergo significant epigenetic modifications (53). Even after the stimulus diminishes, certain epigenetic changes remain, enhancing gene transcription upon subsequent stimulation, thereby bolstering the non-specific immune response (20), This process involves the regulation of histone modifications, DNA methylation, and non-coding RNA s(ncRNAs) (54).

In trained immunity, key histone modifications include the enrichment of H3K4me3 at promoter regions, the accumulation of H3K4me1 at distal enhancer regions, and enrichment of histone 3 lysine 27 acetylation (H3K27ac) in both promoters and enhancers (55, 56). Among these, H3K27ac gradually diminishes after the stimulus ceases, while H3K4me1/3 persists, maintaining long-term cellular hyper-reactivity (57). Furthermore, H3K4me3 is found in the promoter regions of glycolysis-related genes (e.g., hexokinase) in trained monocytes, linking epigenetic modifications directly to metabolic reprogramming (39). The reduction of repressive marks further enhances chromatin accessibility, thereby facilitating trained immunity (40, 42). Recently, histone lactylation has been identified as a metabolic modification that plays a critical role in regulating trained immunity (44).

Epigenetic enzymes also play pivotal roles in trained immunity; Set7 and lysine methyltransferase G9A mediate the “writing” of H3K4me1 and H3K9me2, respectively (52, 57), while KDM4, KDM5, and Sirtuin-1 deacetylases form the “erasure system”, removing H3K4me3, H3K9me3, and H3K27ac marks (42, 58, 59). While the core function of histone modifications are well understood, the precise targeting of specific genomic regions remains unclear.

Recent studies have also emphasized the significant role of ncRNAs in integrating metabolic and epigenetic regulation within trained immunity. In the β-glucan-trained monocyte model, microRNAs (miRNAs), such as miR-9-5p, suppress the expression of the metabolic enzyme isocitrate dehydrogenase 3α (IDH3α), reducing α-KG levels and promoting fumarate accumulation. This metabolic shift enhances inflammation through two mechanisms: First, fumarate stabilizes HIF-1α, driving glycolysis and pro-inflammatory cytokine secretion. Second, fumarate inhibits histone demethylase KDM5, increasing H3K4me3 modification at the promoters of pro-inflammatory genes, enhancing chromatin accessibility and transcription activation. Monocytes deficient in miR-9-5p show a metabolic shift toward OXPHOS, alongside reduced H3K4me3 levels and impaired anti-infection capacity. These results indicate that miR-9-5p is a critical link between metabolic reprogramming and epigenetic remodeling (60).

Additionally, long non-coding RNAs (lncRNAs) that regulate immune gene promoters(IPLs) achieve precise epigenetic reprogramming by altering the chromatin’s three-dimensional structure. For instance, the upstream master lncRNA of inflammatory chemokine loci (UMLILO) recruits the histone methyltransferase MLL1 to the CXCL gene promoter within a topologically associated domain (TAD), enriching H3K4me3 and promoting transcription (61). Mouse models have shown that deleting UMLILO prevents the trained immunity phenotype induced by β-glucan, whereas its insertion into the chemokine TAD restores immune memory function, underscoring its necessity (61). In the β-glucan model, UMLILO activation is dependent on the nuclear factor of activated T cells, and many IPL promoters contain multiple transcription factor binding sites, which could explain how different stimuli activate common pathways. Although the specific deposition of IPLs may contribute to the specificity of epigenetic modifications, studies so far have focused solely on the β-glucan stimulation model, and their applicability to other immune stimulations remains uncertain. Notably, miRNAs and IPLs may miRNAs and IPLs may work together within a regulatory network, where miR-9-5p indirectly influences chromatin accessibility through the metabolic-epigenetic axis, while IPLs such as UMLILO directly reconfigure chromatin structure, jointly maintaining the long-term hyper-reactivity of pro-inflammatory genes. Future studies should explore the interactions among ncRNAs, metabolic intermediates, and chromatin modification enzymes to elucidate the dynamic mechanisms that govern epigenetic memory in trained immunity.

The role of DNA methylation in trained immunity requires further investigation. Preliminary studies suggest that, in the β-glucan-induced monocyte trained immunity model, DNA methylation pattern remain largely unchanged, while LPS-induced immune tolerance is associated with stable, long-term DNA methylation modifications, indicating that DNA methylation may primarily regulate gene expression inhibition (56). Clinical research has shown that the DNA methylation changes induced by BCG vaccination in neonatal monocytes can persist up to 14 months post-vaccination (62). In adult vaccine recipients, responders show widespread loss of DNA methylation at inflammatory gene promoters. Genome-wide analysis has identified significant methylation differences at 43 loci, which may serve as biomarkers for predicting individual responses to trained immunity (63, 64). These findings suggest that DNA methylation may be crucial in the establishment and maintenance of immune memory through dynamic modifications.

In conclusion, during trained immunity, the metabolic reprogramming of innate immune cells connects various metabolic pathways. Metabolic intermediates act as substrates or cofactors for epigenetic enzymes, linking metabolic alterations to the expression of inflammatory genes via epigenetic reprogramming.

## 3 Trained immunity in diabetes

Chronic low-grade inflammation associated with diabetes is a critical driver of vascular lesions (65). Even when metabolic parameters, such as blood glucose, are controlled, prior metabolic disturbances can perpetuate increased cardiovascular risk through the mechanism of “metabolic memory” (11). Recent studies show that diabetes induces “trained immunity” in innate immune cells via epigenetic and metabolic reprogramming, thereby sustaining chronic inflammation (66). Specifically, pathological factors such as hyperglycemia, dyslipidemia, obesity, and unhealthy lifestyle habits remodel the epigenetic landscape of HSPCs, leading to the persistent activation of pro-inflammatory pathways in innate immune cells (12). This immune memory enables these cells to maintain elevated glycolytic activity and increased secretion of inflammatory mediators, even after removal from a metabolically imbalanced environment, ultimately accelerating the progression of vascular diseases (67).

### 3.1 Hyperglycemia

In diabetic patients, the mechanism underlying trained immunity is complex. Hyperglycemia can induce monocytes and macrophages to establish innate immune memory. Specifically, human primary monocytes pretreated with 25 mmol/L high glucose *in vitro* exhibit increased secretion of IL-6 and TNF-α, along with enhanced glycolysis upon subsequent LPS re-stimulation, even after being cultured under normal glucose conditions for 5 days. This process is mediated by MLL family-dependent H3K4me3 epigenetic modification (68). Clinical evidence reveals that H3K4me3 and H3K9ac modifications at pro-inflammatory gene loci in monocytes from diabetic patients are abnormally enriched, confirming that metabolic memory sustains the pro-inflammatory phenotype via epigenetic modifications over an extended period (69). Similarly, neutrophils are influenced by hyperglycemia, where metabolic reprogramming leads to coenzymeA accumulation, triggering histone acetylation and promoting excessive formation of neutrophil extracellular traps (NETs). This phenomenon not only intensifies inflammatory responses but also delays wound healing in diabetic patients (70).

The long-term influence of hyperglycemia on the immune system is largely attributed to its effect on HSPCs. In streptozotocin-induced diabetic mouse models, hyperglycemia drives bone marrow hematopoiesis by upregulating S100A8/A9, leading to an increase in circulating neutrophils and Ly6-C(hi) monocytes, thereby accelerating AS progression (71). Further studies have revealed that hyperglycemic exposure can induce the accumulation of Set7-mediated H3K4me1 marks in the NF-κB-p65 promoter region of HSPCs. The monocytes derived from these cells display significantly elevated IL-6 and TNF-α secretion upon LPS stimulation, and this pro-inflammatory phenotype persists even after blood glucose normalization (72). HSPCs in the bone marrow of patients with T2D also exhibit upregulated expression of pro-inflammatory genes, and the monocytes derived from these cells have accumulated H3K4me1 marks at the NF-κB-p65 promoter, validating that hyperglycemia can convey immune memory across generations through epigenetic “imprinting” (72).

Non-immune cells also exhibit hyperglycemic memory. Vascular smooth muscle cells(VSMCs) in diabetic mice manifest a pro-atherosclerotic phenotype, characterized by enhanced migratory capacity and sustained high expression of pro-inflammatory genes (73). VECs are highly responsive to blood glucose fluctuations: short-term hyperglycemia induces sustained inflammatory responses by increasing H3K4me1 modification in the promoter regions of pro-inflammatory genes, with this effect remaining irreversible even after subsequent blood glucose normalization (74). This epigenetic memory closely resembles the mechanism of trained innate immunity, suggesting that vascular cells and the immune system form a synergistic pro-inflammatory network under metabolic stress.

Short-term blood glucose fluctuations, such as transient intermittent hyperglycemia (TIH), also exert significant pathological effects. In Apolipoprotein E knockout mouse models (*ApoE-/-*), TIH accelerates AS progression by increasing circulating monocytes and neutrophils via activation of bone marrow hematopoiesis. This process depends on the binding of S100A8/A9 to the receptor for advanced glycation end products (AGEs) on myeloid progenitor cells (75). Additionally, the interaction between AGEs and their receptors can activate macrophage inflammatory responses, creating a pro-inflammatory positive feedback loop (76). Notably, TIH does not affect HbA1c levels, suggesting that traditional blood glucose monitoring may underestimate the risk of metabolic memory. Thus, an integrated assessment system combining continuous glucose monitoring with epigenetic markers is urgently required.

Hyperinsulinemia associated with insulin resistance might enhance the secretion and migratory capacity of inflammatory factors in monocytes via the PI3K/Akt/mTOR pathway (77), though insulin itself may inhibit NF-κB activation in a hyperglycemic environment (78), illustrating the complexity of its immunomodulatory actions. While insulin may play a role in regulating trained immunity, its precise mechanism remains to be fully elucidated. Furthermore, hyperglycemia-induced lactate accumulation in diabetic patients may mediate the activation of trained immunity via histone lactylation modifications.

The above findings demonstrate that hyperglycemia remodels chromatin accessibility through metabolic reprogramming and histone modification, keeping pro-inflammatory genes in a pre-activated state and establishing trained immunity. This metabolic and epigenetic reprogramming mechanism offers a novel and insightful perspective on the pathological progression of chronic complications in diabetes.

### 3.2 Lipid metabolism disorders

In metabolic disorders like diabetes, lipid metabolism abnormalities are not only a byproduct of abnormal glucose metabolism but also a significant pathological foundation that drives chronic inflammation and AS (10). Recent studies show that aberrant lipid metabolites, such as oxLDL and Lp(a), can induce “trained immunity” in innate immune cells, establishing a persistent low-grade inflammatory state and accelerating vascular lesion progression (30, 79).

*In vitro* experiments have demonstrated that monocytes exposed to oxLDL exhibit sustained glycolytic activity and enhanced secretion of pro-inflammatory cytokines even after the stimulus is removed, alongside enrichment of H3K4me3 marks at pro-inflammatory gene promoters (21). This process involves activation of the mTOR/HIF-1α axis via Toll-like receptors (TLR4/2), upregulation of key glycolytic enzymes, disruption of cholesterol homeostasis through enhanced expression of scavenger receptors (CD36/SR-A), and downregulation of efflux transporters like ABCA1 and ABCG1, which ultimately promotes foam cell formation (80, 81). Moreover, mitochondrial reprogramming induced by oxLDL—characterized by accumulation of TCA cycle intermediates, reactive oxygen species (ROS) production, and enhanced OXPHOS—amplifies the inflammatory response (81).

Clinical evidence confirms that monocytes from patients with familial hypercholesterolemia exhibit a trained immunity phenotype, with elevated H3K4me3 levels, enhanced glycolysis-related gene expression, and persistence of cytokine hyper-reactivity despite 3 months of statin therapy reducing LDL-C levels (82). Analysis of bone marrow progenitor cells reveals that a high-lipid microenvironment induces myeloid-biased differentiation of hematopoietic stem cells (HSCs), with progenitors retaining pro-inflammatory transcriptional signatures even after lipid normalization (83).

Lp(a), the main carrier of oxidized phospholipids (OxPL), contributes to trained immunity through its pro-inflammatory effects (84). *In vitro* studies show that co-incubating healthy human monocytes with plasma from patients with elevated Lp(a) for 24 hours followed by restimulation with Pam3Cys significantly enhances IL-6 and TNF-α secretion. This effect can be blocked by anti-OxPL antibodies, confirming OxPL as the key mediator of Lp(a)-induced trained immunity (85). Clinical observations further reveal that monocytes from patients with elevated Lp(a) exhibit exaggerated pro-inflammatory cytokine secretion and maintain a persistently activated state upon *in vitro* stimulation (85), suggesting that Lp(a) may establishes immune memory through epigenetic or metabolic reprogramming mechanisms.

### 3.3 Obesity

In diabetes, metabolic memory is not only linked to obesity-related metabolic disorders but is also directly induced by obesity itself. Obesity can perpetuate metabolic memory, thereby exacerbating disease progression through the continuous reprogramming of the immune system. Insulin resistance, in conjunction with an unhealthy lifestyle characterized by a high-sugar, high-fat diet (HFD) and physical inactivity, plays a pivotal role in promoting obesity in diabetic patients. Despite successful weight loss, around 80% of patients experience weight regain, which leads to a worsening of metabolic complications (86). Research has demonstrated that chronic inflammation in adipose tissue during obesity is marked by the infiltration of pro-inflammatory macrophages. Although weight loss partially alleviates this inflammation, key immune markers remain elevated, creating an “obesity memory” (87). This long-term alteration in the phenotype and function of immune cells triggered by obesity may underlie the recurrence of metabolic complications upon weight regain.

The formation of obesity-related metabolic memory involves a synergistic interaction between metabolic reprogramming and epigenetic remodeling. Metabolic disturbances in fatty acid metabolism among obese individuals may lead to the accumulation of palmitic acid, a saturated fatty acid associated with various metabolic dysfunctions. Studies have shown that macrophages treated with palmitic acid or secretions from obese adipose tissue exhibit increased glycolysis and OXPHOS activity, along with enhanced secretion of TNF-α and IL-6 upon LPS re-stimulation (88). This process is mediated by the activation of the TLR4/mTOR pathway and epigenetic modifications such as H3K4me3, catalyzed by histone methyltransferases. Inhibition of mTOR or methyltransferases can reverse this “trained” effect, confirming that the interaction between metabolic and epigenetic networks represents the core regulatory mechanism (88). Further investigations have shown that, in comparison to persistent obesity, weight cycling (repeated cycles of weight loss and regain) in animal models induces more severe metabolic dysregulation. The underlying mechanisms include an enhanced lipid-handling capacity of adipose tissue macrophages, hyperactivation of antigen-presenting cells, and T cell exhaustion (89). Under these conditions, metabolic memory amplifies inflammation through a positive feedback loop, leading to sustained chemokine release from adipose tissue and recruitment of pro-inflammatory monocytes, further disrupting metabolic homeostasis.

A growing body of evidence supports the existence of obesity-related immune memory. For example, upon *in vitro* stimulation, monocytes isolated from obese patients exhibit significantly greater cytokine secretion capacity compared to those from normal-weight controls, indicative of a baseline activated state (90). Specifically, *in vitro* LPS stimulation triggers higher levels of IL-1β, IL-6, IL-8, and TNF production by monocytes derived from obese patients (90). Moreover, elevated serum leptin levels in obese patients correlate with higher IL-1β and IL-6 concentrations. *In vitro* experiments further confirm that monocytes pretreated with leptin demonstrate an enhanced inflammatory cytokine secretion capacity upon subsequent LPS re-stimulation (91). Collectively, these findings suggest that monocytes from obese patients possess an intrinsic pro-inflammatory tendency, potentially associated with a memory mechanism akin to trained immunity.

Obesity also drives myeloid-biased differentiation via the fat-bone marrow axis. Similar to the effects of hyperglycemia on bone marrow, obese adipose tissue releases S100A8/S100A9 and stimulates IL-1β secretion from bone marrow progenitor cells via activation of TLR4/MyD88 signaling and NLRP3 inflammasomes, promoting the expansion of monocytes and neutrophils (92). Notably, even after repeated bone marrow transplants, HSCs in obese mice still preferentially differentiate into inflammatory adipose tissue macrophages, an effect that depends on MyD88 signaling (93). This finding reveals the underlying mechanism of persistent immune memory following weight normalization.

### 3.4 Dietary stimulation

Unhealthy dietary habits, such as those associated with the Western diet (WD), are commonly observed during the progression of diabetes. WD is characterized by high levels of sugar, calories, fat, and salt, as well as low fiber and mineral content. It not only contributes directly to obesity, metabolic syndrome, and disorders in glucose/lipid metabolism but also remodels immune homeostasis via multiple mechanisms (94).

Studies have shown that WD and its metabolites can exacerbate chronic inflammation-related diseases by inducing trained immunity. WD can alter the composition of the gut microbiota, decrease protective metabolites, and increase intestinal permeability, allowing LPS to translocate into the circulatory system and trigger metabolic endotoxemia. In *ApoE* ^-^/^-^ mice, LPS-simulated metabolic endotoxemia upregulates miR-24, enhances monocyte inflammatory reprogramming, and accelerates AS progression (95). Notably, low-dose LPS (≤10 pg/mL) induces trained immunity in monocytes, characterized by sustained high expression of pro-inflammatory factors such as TNF-α and IL-6, while high-concentration LPS induces immune tolerance, revealing a dose-dependent regulatory mechanism (96).

In an AS-susceptible model (*Ldlr* ^-^/^-^mice), WD activates the NLRP3 inflammasome and downstream IL-1β signaling, driving transcriptional and epigenetic reprogramming iv granulocyte-monocyte progenitor cells (GMPs) (97). Even after reverting to a normal diet for 4 weeks, myeloid cells retain a hyper-inflammatory phenotype, accompanied by DNA hypomethylation in genes and the expansion of circulating monocyte (97, 98).

A comparative study of urban and rural populations in Tanzania further supports the immunomodulatory effects of WD. Urban residents, whose diet is deficient in flavonoids like apigenin, produce more pro-inflammatory factors in monocytes upon LPS stimulation. In contrast, the high concentration of apigenin in traditional rural diets partially inhibits trained immunity and reduces the inflammatory response (99). Additionally, apigenin blocks oxLDL-induced monocyte trained immunity *in vitro*, suggesting its potential anti-inflammatory properties. In a HFD-induced C57BL/6J mouse obesity model, even when body weight normalizes, bone marrow monocytes and adipose tissue macrophages persist with a pro-inflammatory phenotype (100). Mechanistically, long-term alterations in chromatin accessibility at activator protein 1 (AP-1) binding sites are critical, and this phenomenon can be recapitulated in mouse macrophages through short-term exposure to stearic acid, indicating that some nutrients can directly drive epigenetic reprogramming (99). Moreover, irregular diets are also associated with trained immunity. For example, intermittent HFD increases the number of neutrophils in peripheral blood by reprogramming neutrophil progenitor cells, eliciting a stronger inflammatory response compared to continuous HFD (101).

### 3.5 Gut microbiota dysbiosis

In diabetic patients, the GM typically exhibits reduced diversity, an increase in pro-inflammatory bacteria, and a significant decline in commensal bacteria that produce (SCFAs) (102). This reduction in SCFAs contributes to disturbances in glucose and lipid metabolism, impaired intestinal barrier function, enhanced endotoxin translocation into the bloodstream, and the induction of chronic low-grade inflammation, all of which promote insulin resistance (103). Metabolic abnormalities, such as hyperglycemia and lipid metabolism disorders, and endotoxemia have been shown to trigger trained immunity via epigenetic reprogramming, as discussed previously.

One key metabolite of gut microbiota dysbiosis—trimethylamine N-oxide (TMAO)—plays a crucial role in trained immunity. TMAO is generated by intestinal microbiota through the metabolism of dietary choline and carnitine. Diabetic individuals, due to their tendency to consume high-fat and high-red meat diets (which are rich in choline and carnitine) (104), provide an abundant substrate for TMAO production. TMAO induces endothelial trained immunity by activating endoplasmic reticulum (ER) stress (105). Research has shown that TMAO utilizes PERK (protein kinase R-like ER kinase) as a conditional DAMP receptor (106), triggering ER stress in human aortic endothelial cells (HAECs). This leads to PERK phosphorylation and the activation of the downstream transcription factor cAMP response element-binding protein (CREB), linking ER stress signals with mitochondrial dysfunction and enhanced glycolytic metabolism via the PERK-CREB signaling axis (106). As a result, HAECs adopt a pro-inflammatory phenotype, similar to innate immune cells, resulting in the upregulation of adhesion molecules such as ICAM-1 and inflammatory factors, thereby maintaining a chronic pro-inflammatory state (107, 108). In summary, TMAO induces trained immunity in HAECs through the PERK-CREB axis, involving multiple regulatory mechanisms, including ER stress and metabolic reprogramming. This highlights TMAO as a potential therapeutic target for preventing and treating AS.

Chronic low-grade inflammation, common in diabetes, accelerates vascular lesions through trained immunity. Pathological processes such as hyperglycemia, lipid metabolism disorders, gut microbiota dysbiosis, and obesity induce a persistent pro-inflammatory phenotype in immune cells—especially innate immune cells—through epigenetic and metabolic reprogramming.

## 4 Trained immunity in AS

AS is characterized by chronic inflammation of the arterial intima, which is central to its pathology. The onset, progression, and thrombotic complications of AS are closely linked to the abnormal activation of the innate immune system (109). Trained immunity has a dual effect: while it enhances the host’s resistance to infections via broad-spectrum immune responses, excessive activation leads innate immune cells to form persistent pro-inflammatory phenotypes, exacerbating chronic inflammatory diseases such as AS (14, 110).

Monocytes and macrophages, as the core myeloid cells in AS lesions, can adopt pro-inflammatory and pro-AS phenotypes upon exposure to microbial stimuli or endogenous metabolites (80). Microbial stimulation activates trained immunity by upregulating the transcription of pro-inflammatory genes (111), while endogenous stimuli like oxLDL promote foam cell formation and sustained inflammation through metabolic reprogramming and epigenetic modifications (21). Clinical studies indicate that monocytes in AS patients exhibit trained immunity traits, including enhanced secretion of pro-inflammatory cytokines, increased glycolytic activity, and altered epigenetic/metabolic markers, maintaining heightened reactivity even after *in vitro* differentiation into macrophages (112, 113). Notably, myocardial infarction induces trained immunity in monocytes, thereby increasing the risk of AS-related cardiovascular events (114). Recent studies have demonstrated that type I interferons (IFNs) in sicca syndrome induce monocytes to secrete pro-AS cytokines and enhance cholesterol uptake. This interaction with trained immunity is bidirectional: trained immunity modulates type I IFN production and the transcriptional response to receptor restimulation (115).

In addition to monocytes/macrophages, other myeloid cells such as neutrophils (116) and DCs (117), have been implicated in AS-related immune memory. BCG vaccination induces long-lasting pro-inflammatory phenotypes in neutrophils, upregulating activation markers and enriching H3K4me3 modifications across the genome, which boosts their antibacterial functions (118). Neutrophils from β-glucan-pretreated mice exhibit significantly increased IL-6 secretion, myeloperoxidase production, and resistance to Listeria monocytogenes upon LPS stimulation (119), demonstrating the reprogramming of an anti-tumor phenotype via type I IFN signaling and ROS-mediated mechanisms. This reprogramming effect can be transferred to naïve mice via neutrophil adoptive transfer or bone marrow transplantation, independent of the adaptive immune system (120). Periodontitis, through trained immunity, may elevate neutrophil numbers and pro-inflammatory responses, increasing AS risk (121). However, the role of trained immunity in DCs dendritic cells in AS remains unclear (117).

The persistence of trained immunity depends on the epigenetic and functional reprogramming of bone marrow progenitor cells (122). BCG vaccination triggers transcriptional, epigenetic, and functional reprogramming of HSPCs for up to 3 months, which is then transmitted to peripheral monocytes (123). In AS models, GMPs in *Ldlr-/-* mice undergo continuous reprogramming after 4 weeks of WD feeding, and the differentiated monocytes exhibit a hyper-responsive state under TLR ligand stimulation, which persists even 4 weeks after returning to a conventional diet (97). Clinical studies have shown that HSPC transcriptional profiles in patients with severe coronary artery disease are skewed towards myeloid differentiation, with enrichment of monocyte/neutrophil pathways, although insufficient epigenetic evidence exists to confirm its association with trained immunity (124).

Moreover, non-immune vascular cells may also contribute to AS via trained immunity mechanisms. In VECs, short-term hyperglycemia induces epigenetic remodeling via H3K4me1 enrichment, mediated by Set7 methyltransferase, resulting in sustained high expression of pro-inflammatory genes and maintenance of inflammatory responses even after blood glucose normalization (74, 125). Lysophosphatidylcholine (LPC) activates key trained immunity pathways, inducing metabolic and epigenetic reprogramming in VECs and promoting a persistent pro-atherosclerotic phenotype (126). OxLDL induces prolonged inflammatory factor secretion in HAECs through metabolic reprogramming and histone modification (30).VSMCs can transdifferentiate into macrophage-like cells under certain conditions, promoting AS development. Furthermore, oxLDL or BCG vaccination induces sustained pro-inflammatory responses in VSMCs via metabolic and epigenetic mechanisms, contributing to plaque instability (127). Non-immune cells acquire memory akin to innate immune cells through epigenetic and metabolic reprogramming, collaborating with myeloid cells to drive chronic inflammation in AS. This finding expands the cellular scope of trained immunity and presents new perspectives for targeted intervention.

In conclusion, the pro-atherosclerotic role of trained immunity is increasingly recognized. In AS, effector cells of trained immunity include not only myeloid cells or HSPCs but also vascular non-immune cells. The interactions among these diverse cell types *in vivo* contribute to the onset and progression of AS.

## 5 Trained immunity in diabetes and its role in accelerating AS

**Figure 3:**
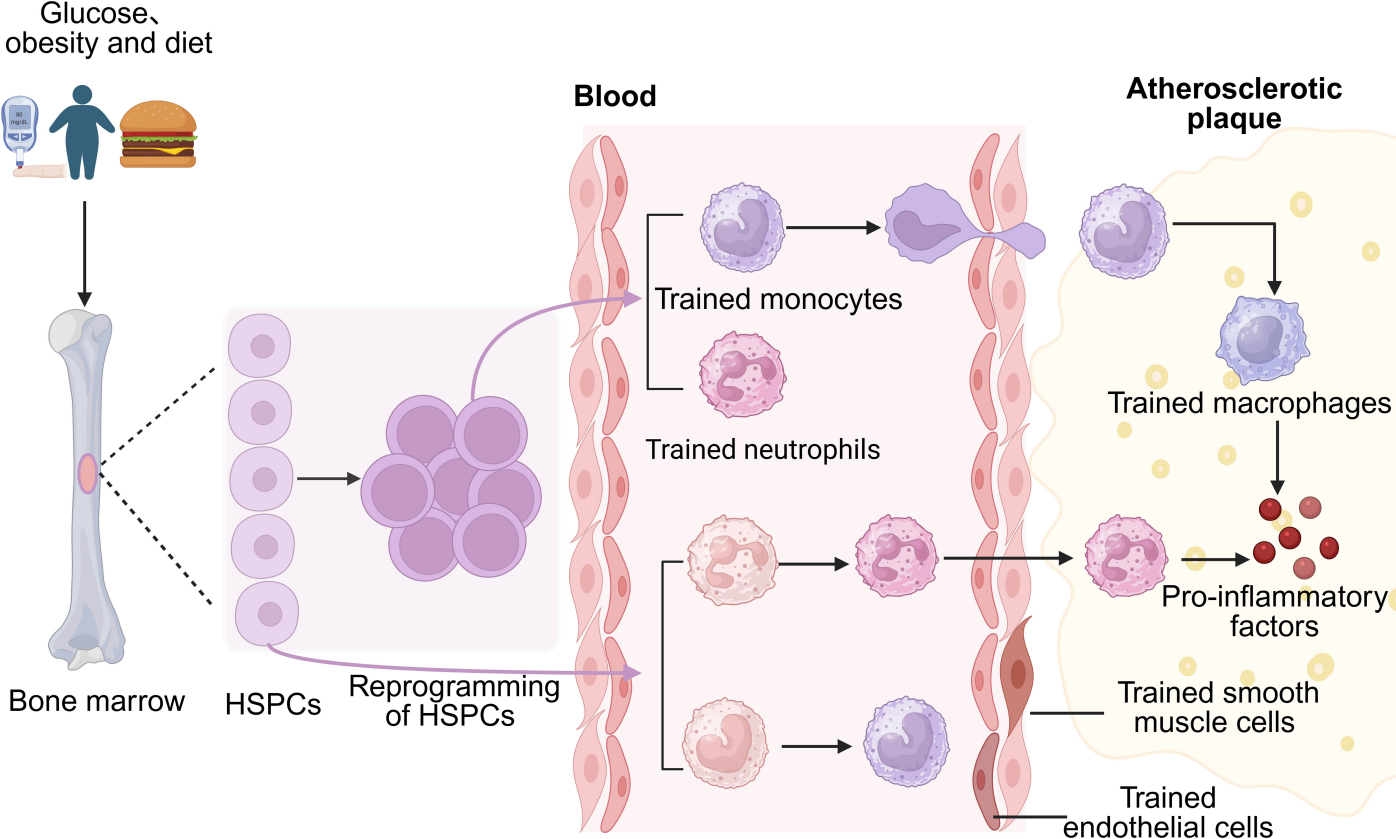
Diabetes reprograms hematopoietic stem and progenitor cells (HSPCs) to induce trained immunity, differentiating into trained monocytes and neutrophils. Normal HSPC-derived monocytes and neutrophils can also be trained. Trained neutrophils infiltrate atherosclerotic plaques directly, while trained monocytes migrate to plaques and transform into macrophages. Both cell types secrete pro-inflammatory factors, promoting plaque formation. Additionally, trained immunity can occur in non-immune vascular cells, such as endothelial cells.

Diabetes-induced pathological changes can significantly contribute to complications such as AS. Despite therapeutic interventions aimed at regulating blood glucose and correcting other metabolic disturbances, patients’ cardiovascular risks often persist—this phenomenon is referred to as “metabolic memory” (11). Studies indicate that the long-term impact of diabetes on innate immune cells remains largely irreversible even after metabolic intervention, suggesting that the mechanisms involved extend beyond mere metabolic regulation. Recent research has revealed that diabetes-induced epigenetic reprogramming in the bone marrow niche locks myeloid cells into a pro-inflammatory state via trained immunity, which continues to drive AS progression even after metabolic abnormalities are corrected (128), thus offering a critical molecular explanation for “metabolic memory” (**Figure 3**).

To explore the connection between hyperglycemia, trained immunity, and AS, a study using streptozotocin to induce diabetes in mice conducted a comprehensive investigation (16). The findings showed that bone marrow cells from diabetic mice, when differentiated into BMDMs under normal blood glucose conditions and stimulated, demonstrated significantly increased IL-6 expression, alongside enhanced uptake of modified LDL and foam cell formation—hallmarks of a pro-AS phenotype. When bone marrow from diabetic mice was transplanted into *Ldlr-/-* mice with normal blood glucose levels and placed on a WD, AS progression accelerated dramatically. Mechanistically, high-glucose culture of HSCs led to the upregulation of H3K4me3 and H3K27ac modifications, which are associated with glycolytic metabolic reprogramming. Macrophages within the aortic plaques of these mice displayed enriched H3K4me3 modification, indicating that hyperglycemia promotes trained immunity via epigenetic memory in progenitor cells, a process that is transmittable across generations. Additional studies demonstrated that this mechanism depends on the regulation of the transcription factor Runx1 (16). Importantly, similar epigenetic and transcriptional changes were observed in peripheral blood mononuclear cells and AS plaque macrophages from diabetic patients, confirming the presence of a hyperglycemia-induced trained immunity mechanism in human diabetic AS (16).

Another study focused on the epigenetic regulator histone deacetylase 3 (HDAC3), revealing that its expression in plaque macrophages of diabetic AS patients was significantly elevated and positively correlated with serum LDL, triglycerides, and blood glucose levels (129). *In vitro* experiments demonstrated that in a high-glucose environment, LPS stimulation markedly increased glucose uptake and lactate/succinate production in BMDMs, promoting polarization toward the pro-inflammatory M1 phenotype and enhancing secretion of IL-6, IL-1β, and TNF-α, which facilitated foam cell transformation and AS plaque progression. Animal studies further showed that specific knockout of HDAC3 in macrophages mitigated AS lesions in high-fat-diet (HFD)-fed *ApoE-/-* mice, indicating that HDAC3 exacerbates AS progression by mediating metabolic dysregulation and epigenetic modifications in macrophages (129). While these observations suggest a role for trained immunity, the exact regulatory patterns of epigenetic changes on specific target genes and their temporal effects remain unclear. Future studies should investigate the persistent association between pro-inflammatory phenotypes and epigenetic modifications to elucidate the molecular mechanisms of HDAC3 in diabetes-associated AS.

Diet-induced trained immunity is another significant factor driving AS progression. A HFD enhances the pro-inflammatory activity and abundance of circulating neutrophils in *Ldlr-/-* mice by reprogramming neutrophil progenitor cells, thereby accelerating AS development (101). In AS model mice fed a WD for 4 weeks, granulocyte-monocyte progenitors (GMPs) and differentiating monocytes/macrophages exhibited persistent functional and epigenetic reprogramming. Notably, even after reverting to a normal diet, these myeloid cells retained a highly inflammatory phenotype (97). Further experiments demonstrated that bone marrow transplantation from WD-fed mice into *Ldlr-/-* mice on a regular diet significantly increased AS plaque size, independent of serum cholesterol levels. This effect was closely linked to abnormal DNA methylation in myeloid cells and the expansion of circulating monocytes (98). Another study demonstrated that HDAC9 expression was significantly upregulated in plaque macrophages of WD-fed *Ldlr-/-* mice. HDAC9 reduced histone 3 acetylation, inhibiting the expression of ABCA1, ABCG1, and the nuclear receptor PPARγ, thus impairing cholesterol efflux and promoting macrophage polarization toward the M1 phenotype. In contrast, HDAC9 deficiency enhanced cholesterol efflux and reduced inflammatory mediators like IL-1β and MCP-1, thereby suppressing AS progression (130). Together with the findings on HDAC3, both regulators affect innate immunity by modulating TLR and IFN signaling pathways, suggesting that HDAC inhibitors could suppress trained immunity activation by targeting these pathways (131).

In obesity-related chronic low-grade inflammation, the adipokine leptin secreted by adipocytes plays a crucial regulatory role through trained immunity. Studies show that brief exposure to physiological leptin concentrations significantly enhances the response of monocytes differentiated *in vitro* to LPS stimulation, increasing TNF-α secretion, phagocytic function, and foam cell formation—processes directly linked to AS pathogenesis (91). Clinical data further support that serum leptin levels in obese individuals correlate positively with inflammatory markers such as IL-6 and IL-1β. Leptin gene polymorphisms also demonstrate gender-specific regulation of IL-6, with more pronounced effects observed in men (91). These findings suggest that leptin continuously activates monocytes/macrophages via trained immunity, driving inflammation and lipid metabolism disorders that accelerate AS progression. Gender differences may influence the molecular regulatory pathways involved. In studies of lipid metabolism disorders, pre-stimulation of monocytes with oxLDL for 6 days followed by re-stimulation with TLR agonists induces sustained enhancement of AS-related pro-inflammatory factor secretion. This phenotype is maintained through glycolysis upregulation and metabolic-epigenetic reprogramming mediated by histone H3K4me3 modifications (21). Similar mechanisms are observed in metabolic endotoxemia induced by gut microbiota dysbiosis and in TMAO-induced ER stress via the PERK pathway, both of which promote AS progression through trained immunity (95, 108).

Recent studies have highlighted that miRNAs play a pivotal role in diabetic AS by targeting metabolic and immune pathways. Specifically, miRNA-425-5p exacerbates endothelial dysfunction by suppressing monocarboxylate transporter MCT4 expression (132). In high-glucose or IL-1β-stimulated human umbilical vein endothelial cells (HUVECs) derived from diabetic patients, the significant upregulation of miRNA-425-5p leads to reduced MCT4 expression, resulting in impaired lactate efflux and intracellular accumulation. This ultimately triggers metabolic disturbances and apoptosis in endothelial cells. These findings suggest that the miRNA-425-5p-mediated imbalance of the glycolysis-lactate axis is a critical factor in diabetic vascular injury. Silencing this miRNA may offer a potential strategy for restoring MCT4 expression and improving endothelial cell function, providing an intervention for diabetic vascular complications (132). Further research has demonstrated that miRNAs contribute to AS progression by regulating immune responses and metabolic reprogramming. For instance, high glucose induces macrophage polarization toward the pro-inflammatory M1 phenotype via miR-33-mediated glycolytic reprogramming, thereby intensifying vascular wall inflammation and plaque instability (133). Conversely, miR-638 inhibits the proliferation, migration, and glycolysis of VSMCs by targeting lactate dehydrogenase A, exhibiting anti-AS potential (134). These two miRNAs regulate AS pathogenesis from both pro-inflammatory and anti-proliferative perspectives. More importantly, miRNAs may serve as a crucial nexus between metabolic abnormalities and epigenetic memory. As previously described, miR-9-5p reshapes the epigenetic landscape of pro-inflammatory and glycolysis-related genes in β-glucan-trained monocytes by suppressing the expression of the metabolic enzyme IDH3α. This indicates that miR-9-5p mediates metabolic reprogramming to regulate epigenetic modifications, thus modulating trained immunity. However, the precise regulatory mechanisms underlying miR-9-5p’s role in diabetes-associated trained immunity remain to be further investigated. Future studies should integrate multi-omics analyses with functional validation to explore how miRNAs coordinate metabolic reprogramming and epigenetic modifications, revealing their global regulatory networks in diabetic AS. The development of specific inhibitors or agonists targeting key miRNAs holds promise for advancing precision therapies for diabetic vascular complications.

In conclusion, even with targeted interventions, trained immunity remains a critical factor in sustaining chronic low-grade inflammation in diabetes and driving AS. This may explain why interventions focusing solely on metabolic disturbances, without addressing inflammation, are limited in reducing cardiovascular risk.

## 6 Preventive and therapeutic modulation of trained immunity

Targeting the chronic inflammation mediated by trained immunity presents a promising strategy for the prevention and treatment of AS and CVDs. Current anti-inflammatory approaches have been shown to suppress trained immunity in AS patients and reduce inflammatory responses (135). Additionally, colchicine, an NLRP3 inflammasome inhibitor, significantly lowers the risk of cardiovascular events in patients with coronary heart disease (136). However, broad-spectrum anti-inflammatory treatments come with increased risks of infections and limited therapeutic effectiveness (137), highlighting the need for more precise interventions that specifically target the mechanisms underlying trained immunity.

The establishment of innate immune memory is driven by metabolic reprogramming and epigenetic remodeling, both of which present potential therapeutic targets. In terms of metabolic regulation, metformin inhibits monocyte immune training by suppressing the mTOR pathway, effectively eliminating the trained immune phenotype induced by oxLDL (138). Statins inhibit monocyte immune training by blocking cholesterol synthesis, although they are ineffective in familial hypercholesterolemia patients who have already developed trained immunity (82). Concerning epigenetic regulation, the histone methyltransferase inhibitor 5’-methylthioadenosine reverses oxLDL-induced histone methylation, abolishing trained immune characteristics such as foam cell formation (139). Inhibition of histone deacetylases (HDACs), such as HDAC3 and HDAC9, can reduce atherosclerotic lesions in AS mouse models, with the class I HDAC inhibitor sodium valproate showing promising effects in alleviating AS in diabetic *ApoE-/-* mice (140).

Since epigenetic and metabolic mechanisms are critical to cellular function and essential for maintaining normal physiological processes, enhancing therapeutic specificity is crucial to minimize side effects in AS treatments. One strategy for achieving precise intervention involves nanotechnology-based delivery systems (141). For example, statin-loaded recombinant high-density lipoprotein (HDL) nanoparticles can specifically target plaque macrophages in AS mice, effectively reducing local inflammation. Similarly, rapamycin (an mTOR inhibitor)-loaded HDL nanoparticles not only block monocyte trained immunity *in vitro* but also continuously suppress it in mouse heart transplant models by targeting HSCs, significantly improving transplant survival rates (142–144). These delivery systems help reduce systemic toxicity by concentrating the drug locally, offering valuable insights for clinical application. Moreover, identifying specific molecular targets is crucial for increasing intervention accuracy. Rather than broadly inhibiting epigenetic enzymes, targeting activation marks in the promoter regions of inflammatory genes (e.g., IPLs) could more precisely suppress pro-inflammatory phenotypes. On the metabolic side, the small molecule 3PO partially inhibits the inducible glycolytic enzyme PFKFB3, blocking oxLDL-induced trained immunity while preserving basal glycolytic function and avoiding the adverse effects of complete metabolic inhibition (143). Additionally, the timing of intervention is pivotal in determining therapeutic efficacy and safety. Short-term interventions during the critical window of trained immunity initiation can help prevent AS complications while maintaining the plasticity of the innate immune system. Dynamic monitoring of immune activation markers and precise regulation during key disease progression stages can minimize long-term interference with normal immune function, thus maximizing therapeutic benefits.

Aberrant activation of trained immunity is a critical factor in the progression of AS, involving pathways such as type IFN signaling and the PI3K-AKT pathway. These key regulatory nodes provide potential therapeutic targets for AS treatment. Studies have shown that the E3 ligase TRIM29 regulates innate immune responses through multiple mechanisms (145). In respiratory viral infections, TRIM29 suppresses type IFN production, diminishing the antiviral activity of alveolar macrophages and airway ECs. Its deficiency significantly enhances IFN secretion and protects mice from influenza infection (146, 147). Under metabolic stress, TRIM29 stabilizes the ER stress pathway by interacting with the PERK protein, promoting PERK-mediated inflammation and oxidative stress; inhibiting PERK disrupts this interaction and alleviates its pathological effects (148). Moreover, TRIM29 inhibits inflammasome activation by ubiquitinating and degrading NLRP6/NLRP9b, reducing IL-18 production, a key pro-inflammatory cytokine (149). These findings suggest that targeting TRIM29 may suppress AS progression through various pathways, including blocking IFN activation, mitigating ER stress, and inhibiting inflammasome activation.

The PI3K-AKT pathway has bidirectional regulatory effects in AS: moderate activation stabilizes plaques by inhibiting NF-κB-mediated inflammatory cytokine production and promoting collagen synthesis, while excessive activation contributes to insulin resistance and abnormal proliferation of VSMCs, increasing the risk of plaque rupture (150). Further research has identified poly(ADP-ribose) polymerase 9 (PARP9), an upstream regulator of this pathway, which induces type I IFN production in DCsdendritic cells and macrophages via the PI3K/AKT3 pathway (151). In the AS microenvironment, sustained activation of immune cells within plaques leads to excessive IFN production, which may induce trained immunity and create a positive feedback loop of chronic pro-inflammatory cytokine release. Targeting PARP9 may inhibit plaque deterioration by modulating PI3K/AKT3 activity and disrupting the IFN-driven inflammatory cascade.

In conclusion, targeting TRIM29 or PARP9 signaling could offer novel therapeutic strategies for the prevention and treatment of AS by inhibiting the formation of trained immunity. However, the specific roles of these targets, such as the dynamic expression patterns of TRIM29 in AS plaques, and the clinical feasibility of translating these findings, including the tolerability and efficacy of PARP9 inhibitors, require thorough validation. Future research should incorporate single-cell multi-omics and dynamic pathological models to clarify their precise regulatory networks in AS, paving the way for the clinical translation of these discoveries into targeted interventions.

## 7 Conclusion and future perspectives

It is projected that in the near future, the incidence of diabetes and its associated AS will continue to increase, particularly in regions with a high prevalence of metabolic syndrome, such as China. While current clinical guidelines emphasize the management of blood glucose and lipid levels, large-scale studies have demonstrated that even diabetic patients with well-controlled metabolic parameters still face significant residual cardiovascular risks. This highlights the need for a shift in treatment paradigms, with the “trained immunity” mechanism driven by diabetes offering a novel approach to address this challenge.

Recent research has revealed that diabetes can substantially accelerate the pathological progression of AS by inducing prolonged activation of trained immunity. This process is characterized by persistent metabolic reprogramming in myeloid cells, dysregulated epigenetic changes, and excessive production of pro-inflammatory mediators. These findings explain why interventions targeting only metabolic disturbances, such as hyperglycemia, are insufficient to effectively mitigate cardiovascular risk, while also emphasizing the potential of precision medicine through the modulation of trained immunity. Possible therapeutic targets include the inhibition of inflammatory signaling pathways, the reversal of aberrant epigenetic modifications, or the modulation of critical metabolic processes. However, current therapeutic strategies face two main challenges: first, the molecular mechanisms underlying trained immunity, such as metabolic reprogramming, are intricately linked to various physiological functions; second, much of the evidence to date relies on *in vitro* monocyte models and animal studies, with limited validation in human clinical data.

To address these challenges, a stratified intervention approach could be employed. This might include the use of nanocarriers for targeted drug delivery to myeloid cells, the development of epigenetic editing technologies for targeting inflammation-related gene loci, and the implementation of short-term interventions during the critical initiation phase of trained immunity. Additionally, a more detailed examination of specific molecular markers (e.g., IPLs) could pave the way for the development of more selective and effective therapeutic strategies. Despite the current translational challenges, targeting trained immunity represents a promising new paradigm for overcoming the “metabolic memory” of diabetic AS and may drive a strategic shift in cardiovascular treatments from focusing solely on metabolic regulation to incorporating immune remodeling.
